# Climate Change, Population Immunity, and Hyperendemicity in the Transmission Threshold of Dengue

**DOI:** 10.1371/journal.pone.0048258

**Published:** 2012-10-29

**Authors:** Mika Oki, Taro Yamamoto

**Affiliations:** Department of International Health, Institute of Tropical Medicine, The Global Center of Excellence, Nagasaki University, Nagasaki, Japan; University of Rochester, United States of America

## Abstract

**Background:**

It has been suggested that the probability of dengue epidemics could increase because of climate change. The probability of epidemics is most commonly evaluated by the basic reproductive number (R_0_), and in mosquito-borne diseases, mosquito density (the number of female mosquitoes per person [MPP]) is the critical determinant of the R_0_ value. In dengue-endemic areas, 4 different serotypes of dengue virus coexist–a state known as hyperendemicity–and a certain proportion of the population is immune to one or more of these serotypes. Nevertheless, these factors are not included in the calculation of R_0_. We aimed to investigate the effects of temperature change, population immunity, and hyperendemicity on the threshold MPP that triggers an epidemic.

**Methods and Findings:**

We designed a mathematical model of dengue transmission dynamics. An epidemic was defined as a 10% increase in seroprevalence in a year, and the MPP that triggered an epidemic was defined as the threshold MPP. Simulations were conducted in Singapore based on the recorded temperatures from 1980 to 2009 The threshold MPP was estimated with the effect of (1) temperature only; (2) temperature and fluctuation of population immunity; and (3) temperature, fluctuation of immunity, and hyperendemicity. When only the effect of temperature was considered, the threshold MPP was estimated to be 0.53 in the 1980s and 0.46 in the 2000s, a decrease of 13.2%. When the fluctuation of population immunity and hyperendemicity were considered in the model, the threshold MPP decreased by 38.7%, from 0.93 to 0.57, from the 1980s to the 2000s.

**Conclusions:**

The threshold MPP was underestimated if population immunity was not considered and overestimated if hyperendemicity was not included in the simulations. In addition to temperature, these factors are particularly important when quantifying the threshold MPP for the purpose of setting goals for vector control in dengue-endemic areas.

## Introduction

Dengue virus infection is caused by any of the 4 dengue viruses transmitted by *Aedes aegypti*. It is estimated that more than one-third of the global population is living in areas endemic for dengue infection [1], and major outbreaks repeatedly occur in the tropics and subtropics.

The probability of dengue epidemics may increase with increasing changes in the climate [2], because higher temperatures increase the competence of *Ae. aegypti* by facilitating its propagation and viral replication [3], [4]. In previous studies, the probability of epidemics has most commonly been evaluated by the basic reproductive number (R_0_) [5]. R_0_ is defined as the expected number of secondary cases produced by the index case in a naive population during the entire period of infectiousness [6]. When R_0_≥1, the transmission maintains and spreads in the population, and when R_0_<1, the transmission declines and ceases. For mosquito-borne diseases including dengue, R_0_ is directly proportional to the mosquito density (the number of female mosquitoes per person [MPP]) if the biting rate of the vector is steady [7]. Thus, the probability of epidemics can be estimated by the threshold MPP that results in R_0_ being ≥1 in non-endemic areas where the whole population can be assumed to be naive to dengue viruses. When a lower MPP results in R_0_≥1, the risk of epidemics is considered to be higher. The effective reproductive number (R) is similar to R_0_ but can account for population immunity [8]. By using R instead of R_0_, the probability of epidemics can also be estimated in areas where only one serotype of dengue viruses is endemic.

However, in dengue-endemic areas where large epidemics have repeatedly occurred, multiple serotypes coexist (a state known as hyperendemicity). Infection by each serotype induces life-long immunity, and a certain proportion of the population is immune to one or more of these serotypes. Since a high prevalence of anti-dengue antibodies in the population (seroprevalence) increases the transmission threshold independent of temperature [9], one possibility is that repeated epidemics sustain seroprevalence at higher levels and reduce the risk of future dengue epidemics. On the other hand, vigorous vector-control measures have been implemented in many endemic areas. If these vector controls effectively reduce the chance of infection, the number of susceptible individuals will increase in the population, and the probability of epidemics may increase in the future. Thus, in the case of dengue, the changing trend in epidemic potential is determined by the complex interaction between mosquito abundance, climatic conditions, the number of serotypes circulating in the area, and the proportion of the population immune to these serotypes.

As achieving the threshold MPP can be the goal for vector-control strategies, if estimated based on the local setting [9], the fluctuation of population immunity and hyperendemicity should be considered when quantifying the threshold MPP in dengue-endemic areas. However, these factors are not fully considered in R_0_ and R calculations. In the present study, we aimed to investigate the effects of temperature change, population immunity, and hyperendemicity on the threshold MPP that triggers an epidemic by using a mathematical model of dengue transmission dynamics_._

## Methods

### The Model

We created a susceptible-exposed-infectious-recovered model of dengue virus transmission in a closed population on the basis of our previous study [10]. The detailed methodology of creating the model including all parameters and their values is presented in Text S1.

### Simulations

#### Area for simulation

Singapore was chosen for our simulation, from the current dengue-endemic areas.

#### Climate data

Monthly mean temperatures in the 1980s (1980–1989), the 1990s (1990–1999), and the 2000s (2000–2009) were obtained [11] and converted into daily values by interpolation. Precipitation and humidity were assumed to be always sufficient for emergence and survival of mosquitoes. Simulations were conducted in a year starting from January 1st.

#### Virus introduction

When calculating R_0_, the pathogen is assumed to be introduced by a single index case on one occasion. However, in real dengue-endemic areas, infected hosts or vectors occasionally enter the system and maintain virus transmission. To simulate the realistic endemic situation, we assumed that each serotype of dengue virus was successively introduced into the population by 0.03 infectious hosts per day (*I_h_visit_*), which is equivalent to a monthly introduction [10].

#### Number of serotypes

The number of serotypes (*n*) was assumed to be 1, 2, and 4. For a simple approximation of the complex dynamics of hyperendemicity, we assumed that each serotype had equivalent infectivity and prevalence in this model. Mosquitoes that are infected by 2 or more serotypes are rare and negligible. The hosts who are susceptible to *n’* serotypes can be infected by *n’*/*n* of the total infectious mosquitoes [12]. We calculated the number of vectors infected by all infectious hosts, and the number of hosts infected by those vectors was defined as new infections. We assumed that people acquired permanent immunity to that serotype and temporary cross-protective immunity to the other serotypes for 60 days (*T_cross_*) after recovering from the previous infection [13].

#### Population immunity

Population immunity (*p_i_*) was the proportion of individuals who possessed dengue antibody against at least one serotypes. As we assumed equivalent prevalence for all serotypes, the initial prevalence of each serotype was equal at *p_i_*. The initial *p_i_* was set at 0–0.8, with increments of 0.1. The initial susceptible populations at seroprevalence *p_i_* were calculated using the equations in Table 1.

**Table 1:** Equations for the initial susceptible populations at seroprevalence *p_i._*

People who are susceptible to	Symbol	Equation
Primary infection	*S_h1_*	(1−*p_i_*) *N_h_*
Secondary infection	*S_h2_*	*p_i_* (1−*p_i_*) *N_h_*
Tertiary infection	*S_h3_*	*p_i_^ 2^* (1−*p_i_*) *N_h_*
Quaternary infection	*S_h4_*	*p_i_^3^* (1−*p_i_*) *N_h_*

*N_h_* is the human population (100,000).

#### Threshold MPP

We defined an epidemic as a seroprevalence increase of 10% from the baseline value in 1 year. This definition is arbitrary, but has often been used in previous studies [9]. When seroprevalence increases by 10% during an epidemic, the peak prevalence is slightly over 1% of the population; this level is considered to be the minimum value for a detectable epidemic [14], [15]. Simulations were started at MPP = 0.01. The value of MPP increased at increments of 0.01 until an epidemic occurred, and that MPP was defined as the threshold MPP. A lower threshold MPP indicated a higher epidemic potential.

### Model Validation

R_0_ of dengue infection is typically calculated by the following equation when the human population is assumed to be in a steady state [16]:
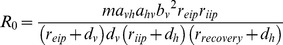
(1)where *m* is the number of female mosquitoes per person, *a_vh_* and *a_hv_* are the transmission probabilities, *b_v_* is the biting rate of adult female mosquitoes, *r_eip_* is the development rate of dengue virus in the vector bodies, *r_iip_* is the development rate of dengue virus in humans, *d_v_* is the mortality rate of adult mosquitoes, *d_h_* is the mortality rate of humans, and *r_recovery_* is the recovery rate of humans. When R_0_ was set at 1, the mosquito density *m* was calculated by rearranging equation 1 as below:



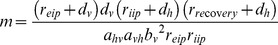
(2)To validate our model, the threshold mosquito density which resulted in R_0_ = 1 (MPP_R0 = 1_) was calculated by our model and compared with *m* under the identical assumption for the simulation. On the basis of the definition of R_0_, we introduced an infectious host (index case) into a naive population on the first day of the simulation. We calculated the number of vectors that were infected by the index case, and the hosts infected by those vectors were defined as secondary cases. The temperature was set at 20°C–35°C and remained constant.

### Uncertainty Analysis

The parameter values we applied in the model were point estimates, but they may vary in nature. Vector mortality (*d_v_*) is one of the important entomological parameters that highly influences the efficiency of virus transmission [17]. We applied *d_v_* = 0.11 (equivalent to an average lifespan of 9.1 days) [18], [19] for the simulation, although the lifespan of *Ae. aegypti* in the field has been estimated to have a relatively broad range, between 5.3 and 9.1 days [20]. The number of blood meals on human beings per gonotrophic cycle (*B*) is also important because it directly determines the frequency of effective contacts with the host. *Ae. aegypti* is known to take multiple blood meals, and therefore, we applied *B* = 2.0 for the simulation. However, the observed frequency of blood feeding by *Ae. aegypti* also widely varies, between once and thrice per cycle [21]. We set transmission probabilities (*a_hv_*: the probability that a susceptible mosquito becomes infected after biting an infectious host, *a_vh_*: the probability that a susceptible host becomes infected by an infectious vector’s bite) to the generally accepted value of 0.75 for both *a_hv_* and *a_vh_* [15], [22], [23]. However, a variety of values has been used for *a_hv_* and *a_vh_* in previous studies, between 0.6 and 1.0 [14], [24], [25]. To assess the effect of these uncertainties on our outcome, we investigated the distribution and the temporal change of threshold MPPs calculated by sampling these parameter values from their possible ranges in the natural environment: 0.11–0.19 for *d_v_*, 1.4–2.0 for *B*, and 0.55–0.95 for both *a_hv_* and *a_vh_*.

### Sensitivity Analyses

We also performed univariate and multivariate sensitivity analyses to evaluate how our findings were affected by each parameter. While conducting sensitivity analyses, the temperature was set at 25°C; the number of serotypes, at 4; and population immunity, at 0%. In univariate sensitivity analysis, we increased the values of *a_hv_*, *a_vh_*, *r_eip_*, *r_iip_*, *r_recovery_*, *B*, *d_v_*, *d_h_*, *T_cross_*, and *I_h_visit_* by 5% and calculated the change of threshold MPP. In multivariate sensitivity analysis, these parameter values were randomly sampled between 25% above and below their baseline values 1000 times. We obtained 1000 estimates of the threshold MPP and conducted multivariate linear regression analysis to investigate the parameters that most strongly affected the model. Significance was set at 0.01. Because our definition of an epidemic was arbitrary, we also changed the definition from a 10% increase of seroprevalence to a 1%, 5%, 15%, and 20% increase of seroprevalence, and the effect on threshold MPP was calculated.

## Results

### Climate Change

**Figure 1:**
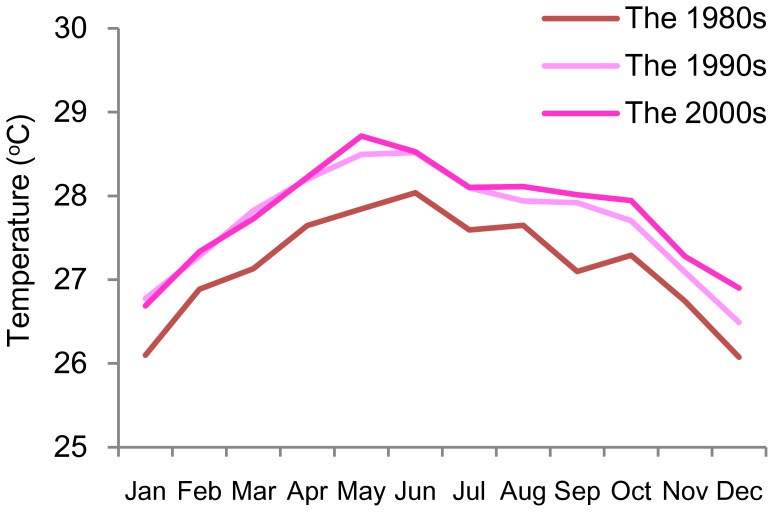
Change in monthly mean temperatures in Singapore.

The change in the monthly mean temperature in Singapore from the 1980s to the 2000s is shown in Fig. 1. Monthly mean temperature in the 1990s was 0.5°C–1.0°C higher than that in the 1980s. There was little change from the 1990s to the 2000s.

### Threshold MPP in Singapore

The threshold MPP in Singapore was investigated as follows:

Simulation 1: Only the effect of temperature was considered.

Simulation 2: Effects of temperature and the fluctuation of population immunity were considered.

Simulation 3: Effects of temperature, fluctuation of population immunity, and hyperendemicity were considered.

#### Simulation 1

**Figure 2:**
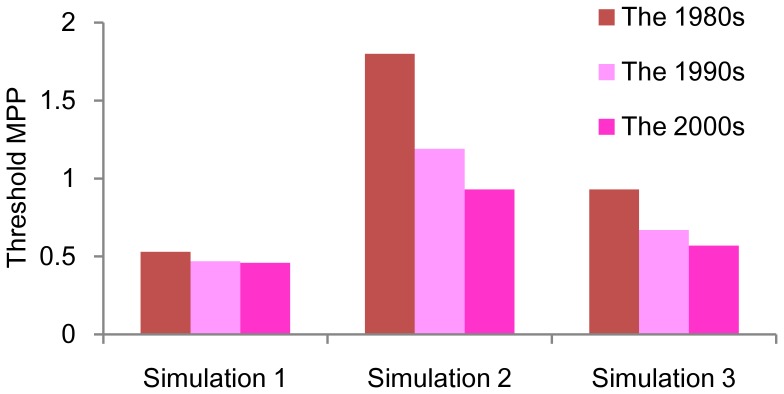
Change in the threshold mosquito density in Singapore from the 1980s to the 2000s. Simulation 1: Effect of only temperature change with single serotype at seroprevalence 0%. Simulation 2: Effects of temperature and the fluctuation of population immunity with a single serotype. Simulation 3: Effects of temperature and population immunity with 4 serotypes. The seroprevalence of dengue antibodies in the Singaporean population was estimated to be 70% in 1980, 60% in 1990, and 50% in 2000 [26]. Threshold MPP is the number of female mosquitoes per person that causes an epidemic (10% increase of seroprevalence). A lower threshold MPP indicates a higher probability of epidemics.

In this simulation, the initial population immunity was assumed to be 0%, and a single serotype was assumed to be involved in the transmission, to limit the scope of the investigation of the effect of temperature. The threshold MPP was estimated to be 0.53 in the 1980s, 0.47 in the 1990s, and 0.46 in the 2000s (Fig. 2). Therefore, the threshold value decreased by 11.3% from the 1980s to the 1990s, but by only 2.1% in the following 2 decades. In total, the threshold MPP decreased by 13.2% during that period.

#### Simulation 2

We additionally incorporated the fluctuation of population immunity in the simulation. A single serotype was involved in virus transmission. The change of seroprevalence (*p_i_*) in the Singaporean population was mathematically estimated in a previous study: the estimated *p_i_* was 0.7 in 1980, 0.6 in 1990, and it reduced to 0.5 in 2000 [26]. If population immunity fluctuated as estimated, the threshold MPP was estimated to be 1.8, 1.19, and 0.93 in the 1980s, 1990s, and 2000s, respectively (Fig. 2). Each value of threshold MPP was more than double that of Simulation 1. In total, the threshold MPP decreased by 48.3% from the 1980s to the 2000s: the decrease was 33.9% during the first 2 decades and 21.9% during the latter 2 decades.

#### Simulation 3

When hyperendemicity (co-circulation of all 4 serotypes) was added into the simulation, the threshold MPP was estimated to be 0.93 in the 1980s, and decreased to 0.67 in the 1990s and to 0.57 in the 2000s (Fig. 2), an overall 38.7% decrease during that period. The decrease was 28% from the 1980s to the 1990s, and 14.9% from the 1990s to the 2000s. The threshold MPPs were lower than those of Simulation 2; however, these values were still higher than those of Simulation 1.

### Overall Effect of Population Immunity and Number of Serotypes

**Figure 3:**
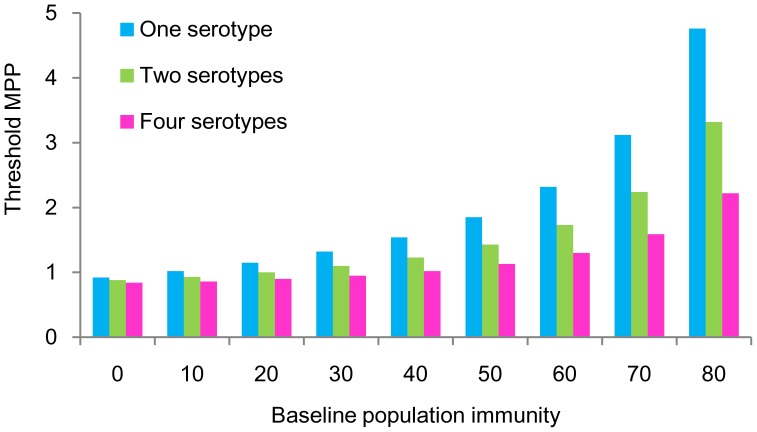
Threshold mosquito density at various population immunity levels and numbers of serotypes. Simulations were conducted at a constant temperature of 25°C. Threshold MPP is the number of female mosquitoes per person that causes an epidemic (10% increase of seroprevalence). A lower threshold MPP indicates a higher probability of epidemics.

We investigated the overall effect of population immunity and the number of serotypes on the threshold MPP. The simulation was performed at a constant temperature of 25°C with various population immunity levels and numbers of serotypes. As shown in Fig. 3, the estimated threshold MPP was lowest (0.84–0.92) at *p_i_* = 0. The threshold MPP increased with an increase in *p_i_*. As compared to a combination of all 4 serotypes, it was found that the estimation of threshold MPP was higher if 1 or 2 serotypes were involved in the transmission. The discrepancy of threshold MPP among numbers of serotypes widened as the population immunity increased (Fig. 3).

### Model Validation

**Figure 4:**
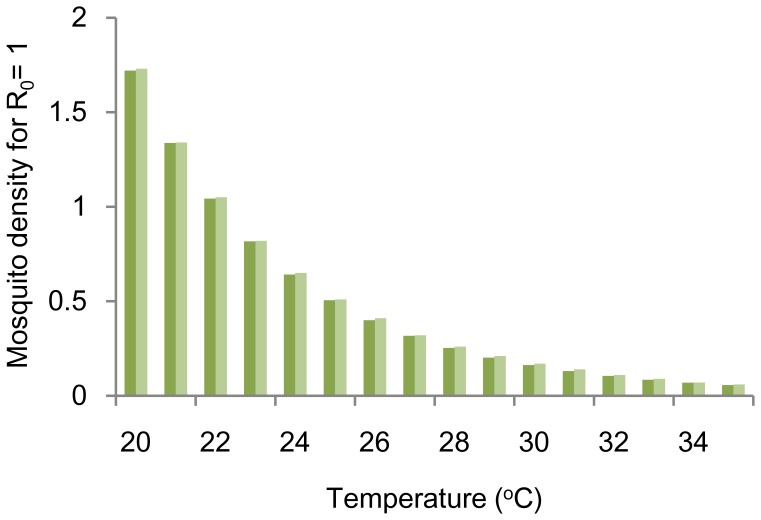
Comparison of the threshold mosquito density resulting in R_0_ = 1. Dark green bars represent *m* calculated by equation 2 [16] and light green bars represent our result (MPP_R0 = 1_).

Our estimation of MPP_R0 = 1_ was very similar to the estimated *m* by equation 2 at all temperatures (Fig. 4). Goodness of fit between our result and *m* was measured by linear regression analysis. The coefficient of determination was 0.9999.

### Uncertainty Analysis

While conducting the uncertainty analysis, the recorded temperatures and the estimated fluctuation of population immunity level in Singapore were applied into the simulation. The number of serotypes was set to 4.

Table 2 shows the values and the temporal changes of threshold MPP by sampling *B*, *d_v_*, *a_vh_*, and *a_hv_* from their possible ranges. The threshold MPP was estimated in the range between 0.58 and 7.32 in the 1980s, 0.42 and 5.26 in the 1990s, and 0.36 and 4.47 in the 2000s. When the most favorable setting for disease spread was assumed, such as a combination of the highest blood feeding frequency (*B* = 2.0), the longest vector survival (*d_v_* = 0.11), and the highest transmission probabilities (*a_vh_*, *a_hv_* = 0.95), the value of threshold MPP was 0.58, 0.42, and 0.36 in the 1980s, 1990s, and 2000s, respectively. These values were 37–38% lower than our base results in Simulation 3 (Fig. 2). When the most unfavorable setting was assumed, such as a combination of the lowest feeding frequency (*B* = 1.4), the highest vector mortality (*d_v_* = 0.19), and the lowest transmission probabilities (*a_vh_*, *a_hv_* = 0.55), the estimated MPP to cause an epidemic was 7.32, 5.26, and 4.47 in the 1980s, 1990s, and 2000s, respectively. These were 6.8–6.9 times higher than our base results in Simulation 3 (Fig. 2). However, the temporal changes in threshold MPP were similar among various parameter settings. The threshold MPP decreased by 27.5–28.4% between the 1980s and 1990s, by 14.3–15.9% between the 1990s and 2000s, and by 37.9–39.1% between the 1980s and 2000s, at any parameter settings (Table 2).

**Table 2:** Result of uncertainty analysis.

*B*	*d_v_*	*a_hv_*, *a_vh_*	Threshold MPP			Change in threshold MPP		
			1980s	1990s	2000s	1980s–1990s	1990s–2000s	1980s–2000s
1.4	0.19	0.55	7.32	5.26	4.47	−28.1%	−15.0%	−38.9%
		0.75	3.94	2.84	2.41	−27.9%	−15.1%	−38.8%
		0.95	2.46	1.77	1.5	−28.0%	−15.3%	−39.0%
	0.15	0.55	5.24	3.77	3.2	−28.0%	−15.1%	−38.9%
		0.75	2.82	2.03	1.73	−28.0%	−14.8%	−38.7%
		0.95	1.76	1.27	1.08	−27.8%	−15.0%	−38.6%
	0.11	0.55	3.49	2.52	2.14	−27.8%	−15.0%	−38.7%
		0.75	1.88	1.36	1.15	−27.7%	−15.4%	−38.8%
		0.95	1.18	0.85	0.72	−28.0%	−15.3%	−39.0%
1.7	0.19	0.55	4.97	3.57	3.04	−28.2%	−14.8%	−38.8%
		0.75	2.68	1.93	1.64	−28.0%	−15.0%	−38.8%
		0.95	1.67	1.2	1.02	−28.1%	−15.0%	−38.9%
	0.15	0.55	3.55	2.56	2.18	−27.9%	−14.8%	−38.6%
		0.75	1.92	1.38	1.17	−28.1%	−15.2%	−39.0%
		0.95	1.2	0.86	0.73	−28.3%	−15.1%	−39.1%
	0.11	0.55	2.37	1.71	1.45	−27.8%	−15.2%	−38.8%
		0.75	1.28	0.92	0.78	−28.1%	−15.2%	−39.0%
		0.95	0.8	0.58	0.49	−27.5%	−15.5%	−38.7%
2.0	0.19	0.55	3.59	2.58	2.2	−28.1%	−14.7%	−38.7%
		0.75	1.94	1.39	1.18	−28.4%	−15.1%	−39.2%
		0.95	1.21	0.87	0.74	−28.1%	−14.9%	−38.8%
	0.15	0.55	2.57	1.85	1.57	−28.0%	−15.1%	−38.9%
		0.75	1.39	1	0.85	−28.1%	−15.0%	−38.8%
		0.95	0.87	0.63	0.53	−27.6%	−15.9%	−39.1%
	0.11	0.55	1.71	1.24	1.05	−27.5%	−15.3%	−38.6%
		0.75*	0.93	0.67	0.57	−28.0%	−14.9%	−38.7%
		0.95	0.58	0.42	0.36	−27.6%	−14.3%	−37.9%

*B* is the number of blood meals on humans per cycle.

*d_v_* is the mortality rate of adult mosquitoes.

*a_vh_* and *a_hv_* are the transmission probabilities.

* Our base results which estimated by our original parameter setting in Simulation 3.

### Sensitivity Analyses

**Figure 5:**
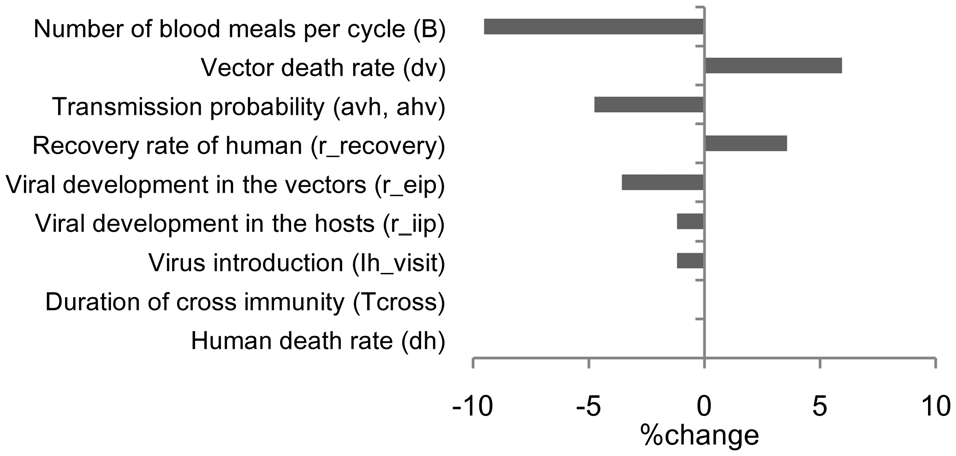
Change in the threshold mosquito density. The effect of a 5% change in each parameter after univariate sensitivity analysis.

The parameters that most strongly affected our outcome were *B*, *d_v_*, transmission probabilities (*a_hv_*, *a_vh_*), and *r_recovery_* in univariate sensitivity analysis; the threshold MPP changed to –9.5%, +6.0%, –4.8%, and +3.6% with 5% increases in the respective parameters (Fig. 5). In the multivariate sensitivity analysis, the parameters that most strongly affected the threshold MPP were similar to those seen in the univariate sensitivity analysis: *B*, *d_v_*, *r_recovery_*, *a_hv_*, *a_vh_*, and *r_eip_*.

When the definition of an epidemic was changed from a 10% increase of seroprevalence to a 1%, 5%, 15%, and 20% increase of seroprevalence, the threshold MPP changed from 0.84 to 0.69 (–17.9%), 0.79% (–6.0%), 0.87 (+3.6%), and 0.89 (+6.0%), respectively.

## Discussion

We used a mathematical simulation model of dengue transmission dynamics that incorporated the effects of climate change, population immunity, and hyperendemicity to estimate the threshold MPP in Singapore. Monthly mean temperature has increased in Singapore in the past 30 years due to climate change, but our results indicated that the impact of this temperature change on the threshold MPP was minor. The fluctuation of population immunity and hyperendemicity more strongly affected the threshold MPP.

In Singapore, the incidence of dengue was successfully reduced in the 1970s by implementing vector-control measures. The premises index (the percentage of premises in which larvae of dengue vector mosquitoes were found divided by the number of premises inspected) was as high as 20%–50% in the 1960s, but it drastically decreased to ∼5% in the 1970s–1980s [27]. Although this entomological index has been maintained at a very low level (approximately 1%–2%) since, major epidemics began to re-emerge in the late 1990s [27]. This phenomenon may be partly explained by a temperature increase due to climate change, which decreased the transmission threshold by 13.2% from the 1980s to the 2000s (Fig. 2). The impact of climate change was primarily observed between the 1980s and the 1990s, with little change between the 1990s and the 2000s.

More importantly, the prevalence of anti-dengue antibodies in the Singaporean population gradually declined through 1980–2000, with a 10% decrease in each decade, as a result of successful vector-control measures implemented in the previous decades [26], [27]. Our simulation showed that the threshold MPP decreased by 38.7% from the 1980s to the 2000s if seroprevalence decreased as estimated (Fig. 2, Simulation 3), and this trend did not change at any settings of the uncertain parameters (Table 2). This result indicated that even if vector density was maintained at a low level, this density eventually reached a new threshold corresponding to the reduced population immunity, and led to an epidemic. Our model showed that population immunity plays a major role in the re-emergence of dengue in Singapore, and that vector population must be further reduced before it reaches a new threshold.

Ideally, serological surveillance is routinely conducted to observe the changing trend in virus-transmission intensity in a population. In the absence of serological assessment, the intensity of viral transmission can be estimated by determining the mean age of infected patients in the endemic situation [26]. A younger mean age of patients would indicate that people are being infected early in life, due to high transmission intensity, and the overall prevalence of dengue antibodies would be high in the population. In Brazil, dengue incidence was previously high in the adult population, but the predominant age group suddenly shifted to a younger generation in 2007 [28]. The ongoing decrease of the mean age of infected persons implies that transmission intensity is increasing, and immediate improvement of the current vector-control strategies is needed. Although dengue is historically classified as a children’s disease in Southeast Asia [29], the mean age of dengue-affected persons has gradually increased in Thailand and Singapore [26], [30]. The increasing mean age of infected persons implies that the current control strategies are successful and that transmission intensity is declining. However, as our simulation in Singapore indicated, reduced transmission lowers population immunity, and more stringent goals for vector control will be needed in the near future. Various local factors may influence the intensity of virus transmission other than climate: mosquito abundance [31], the effect of previous vector control measures [27], the magnitude of viral introduction [32] and introduction of new serotypes [33], the changes in human lifestyle [34], and the demographic transition [35]. Irrespective of the causal factors, because tetravalent dengue vaccines are not yet clinically available, vector control measures are still essential for dengue control. It is important to repeatedly conduct serological and/or epidemiological surveillance, even roughly, to determine the changing trend in transmission intensity in order to design optimal vector-control strategies.

As long as the threshold MPP is quantitatively estimated and utilized to set the goal for vector control measures, we suggest that a certain level of population immunity should be assumed based on the local situation. As shown in Fig. 3, the threshold MPP was always underestimated in our model if we did not consider the population immunity. We also suggest that multiple serotypes should not be simply substituted by single or double serotypes in the simulation. The threshold MPP was not directly proportional to the number of serotypes and was always estimated higher when only 1 or 2 serotypes were assumed to be involved in transmission (Fig. 3). This finding can be interpreted as the transmission threshold being overestimated if calculated with single or double serotypes in areas where all 4 serotypes coexist.

However, our estimation of threshold MPP cannot be directly utilized in the field because there is no qualified entomological index of adult mosquitoes. In practice, the number of pupae per person (PPP) is more frequently used to estimate local mosquito density and the risk of dengue epidemics [36]–[38]. PPP is believed to be the closest approximation for MPP since pupal mortality is relatively low and steady [39], [40]. The transmission threshold for dengue epidemics in terms of PPP was mathematically estimated in a previous study by Focks et al. at constant temperatures (22°C–32°C), assuming that 0%, 33%, and 67% of the population were immune to only one dengue virus [9].

In addition, our sensitivity analyses indicated that some vector parameters such as *B*, *d_v_*, *a_vh_*, and *a_hv_* more strongly affected the value of threshold MPP than other human parameters (Fig. 5). Nevertheless, these entomological parameters remain approximations, because they vary in nature. As we showed in our uncertainty analysis (Table 2), although the magnitude of temporal change in threshold MPP was not much affected by the uncertain parameters, the value of threshold MPP itself was highly affected. The trend shows that the value of threshold MPP increases with a lower biting frequency, a shorter vector lifespan, and lower transmission probabilities. Thus, if our basic assumptions of these parameters are very different from their actual values, the estimated threshold MPP will change. This is one of the limitations of this study.

Our study has a few additional limitations. First, because of the nature of a deterministic model, our model cannot account for stochastic phenomenon in the real-world transmission dynamics.

Second, we assumed that the 4 serotypes of dengue virus have equivalent infectiousness and prevalence in the area. In reality, each virus has different virulence, transmissibility, and behavioral characteristics in the vector bodies [41]–[44]. The dominant serotype can change from year to year [45], and the prevalence of each serotype also fluctuates. If we were to incorporate more complex assumptions of hyperendemicity into our model, the dynamics would change and our findings could be affected.

Third, antibody-dependent enhancement (ADE) is considered to be an important factor in generating a chaotic circulation pattern of multiple serotypes [46], [47]. ADE may operate according to a mechanism by which a pre-existing dengue antibody enhances rather than neutralizes the immune response against a subsequent heterotypic infection [46] and increases the risk of severe clinical manifestation during secondary infection [48]. ADE accelerates viral replication in the host; when the susceptible vector feeds from an infected subject with a high viral load, dengue viruses may be efficiently transmitted to mosquitoes [14]. Thus, ADE may act to reduce the required MPP to cause an epidemic. However, ADE is a negative by-product of hyperendemicity, and our result showed that hyperendemicity itself acts to reduce the threshold MPP (Fig. 3). Therefore, we considered that ADE, if included in our model, would not fundamentally affect our present findings.

Fourth, we employed only ambient temperature in the simulation of the effect of climate change. Various climatic factors other than temperature also affect seasonal mosquito population dynamics. Precipitation may affect breeding-site availability, and humidity may influence mosquito survival [39], [40]. However, in this study, we did not aim to reproduce highly detailed, realistic mosquito population dynamics, but rather aimed to theoretically estimate the threshold MPP and investigate how it is affected by population immunity and hyperendemicity. We assumed that precipitation and humidity were always sufficient for mosquito emergence and survival in our model; *Ae. aegypti* is an extremely domesticated species and primarily breeds in artificial containers in which water availability is independent of rainfall [18]. Thus, we considered that the influence of these climatic factors on our outcome, if any, would be limited.

Fifth, the demographical changes of the human population were not considered in this model. Cummings et al. reported that demographical transitions, such as decreasing birth and death rates in the population, can explain the recent increase in mean age of infected persons in Thailand without any changes of other factors [35]. Because many dengue-endemic countries may experience in the future a change similar to that seen in Thailand, demographical changes will be important factors influencing the epidemic potential of dengue.

Despite these limitations, the present study highlighted the important roles of population immunity and hyperendemicity in the transmission threshold of dengue epidemics. The calculation of R_0_ is useful when we assess the probability of disease spread in a naive population or for diseases that do not induce permanent immunity. However, in the case of dengue, the value of the transmission threshold is strongly affected by factors that are not included in R_0_. We suggest that both population immunity and hyperendemicity should be taken into consideration when quantitatively assessing the threshold vector density for the purpose of setting goals for vector-control strategies in areas where dengue is endemic.

## Supporting Information

Text S1 **The detailed methodology of the model including all equations and parameter values.**
(DOC)
