# Supplementary material for: Climate Change, Population Immunity, and Hyperendemicity in the Transmission Threshold of Dengue
Source: PLoS One. 2012 Oct 29;7(10):e48258. doi: 10.1371/journal.pone.0048258 (PMC3483158; doi:10.1371/journal.pone.0048258)
Supplement: Text S1 — The detailed methodology of the model including all equations and parameter values. (DOC) [file pone.0048258.s001.doc]

**Text S1: Supporting Information**

This supporting information provides the detailed methodology of the model. Values in all equations are daily-based. For the calculation on day *t*, the values from previous day (*t-1*) were taken unless we notified separately. Parameters and parameter values are summarized in Table S1.

**1. Human population**

Human population (*N_h_*) was a closed population (100,000). We did not take age and sex distributions into account. Human life expectancy was arbitrary set to 70 years, thus the human death rate (*d_h_*) was 0.000039. Host population was classified as follows: susceptible hosts (*S_h1_*: people who have never been infected by dengue virus, *S_h2_*: who have gotten primary infection but have a chance to get secondary infection, *S_h3_*: who have gotten infected twice but have a chance to get tertiary infection and *S_h4_*: who have gotten infected three times but have a chance to get fourth infection), exposed hosts (*E_h1_, E_h2_, E_h3_, E_h4_*: people who are infected for the 1^st^ to 4^th^ time, respectively, but not yet infectious to mosquitoes), infectious hosts (*I_h1_, I_h2_, I_h3_, I_h4_*: people who are infected for the 1^st^ to 4^th^ time, respectively and infectious to mosquitoes) and recovered hosts (*R_h1_, R_h2_, R_h3_*: people who have recovered from prior infections) and resistant hosts (*R_h4_*: people who have gotten infected 4 times).

We assumed that people acquire permanent immunity to that serotype after recovering from the previous infection and also possess temporal cross protective immunity (*T_cross_*) to other serotypes for 60 days [1]. The infection with two or more dengue serotypes is considered to produce immunity against following infections and rarely manifest clinical symptoms [2,3]. However, it is still unclear whether people who have got the third or fourth infections possess infectivity to mosquitoes or not. Thus, in our model, we assumed that people were also infectious to mosquitoes during the third or fourth infections.

The number of serotypes (*n*) was set to 1, 2 and 4. We assumed that all serotypes have the identical viral characteristics which were based on dengue virus-2 [4-7] . For a simple approximation of the complex dynamics, it was assumed that all serotypes have the equivalent infectivity and prevalence in this model. Mosquitoes that are infected by 2 or more serotypes are rare and negligible. The hosts who are susceptible to *n’* serotypes can be infected by *n’*/*n* of the total infectious mosquitoes. Thus, we defined *q_1_*- *q_4_* as the proportion of the infectious mosquitoes that can potentially infect the susceptible hosts *S_h1_*- *S_h4_* [8] . The changes in each class of human population are modeled by following differential equations.

(1)

(2)

$\frac{dI_{h1}}{dt}=E_{h1}r_{iip}-I_{h1}(r_{re\mathrm{cov}ery}+d_{h})$ (3)

(4)

(5)

(6)

$\frac{dI_{h2}}{dt}=E_{h2}r_{iip}-I_{h2}(r_{re\mathrm{cov}ery}+d_{h})$ (7)

(8)

(9)

(10)

$\frac{dI_{h3}}{dt}=E_{h3}r_{iip}-I_{h3}(r_{re\mathrm{cov}ery}+d_{h})$ (11)

(12)

(13)

(14)

$\frac{dI_{h4}}{dt}=E_{h4}r_{iip}-I_{h4}(r_{re\mathrm{cov}ery}+d_{h})$ (15)

$\frac{dR_{h4}}{dt}=I_{h4}r_{re\mathrm{cov}ery}-R_{h4}d_{h}$ (16)

Note that *R_h1_* and *R_h2_* were assumed to be resistant hosts while only 1 and 2 serotypes were involved in the transmission, respectively. In those cases, equation 4 and 8 were changed as below:

(4’)

(8’)

Initial population immunity (*p_i_*) was set to 0–0.8 at increment of 0.1. The number of each susceptible population (*S_h1_* – *S_h4_*) was calculated as follows:

$S_{h1}=\left(1-p_{i}\right)N_{h}$ (17)

$S_{h2}=p_{i}\left(1-p_{i}\right)N_{h}$ (18)

$S_{h3}=p_{i}^{2}\left(1-p_{i}\right)N_{h}$ (19)

$S_{h4}=p_{i}^{3}\left(1-p_{i}\right)N_{h}$ (20)

**2. Vector population**

We assumed all mosquitoes were female in our model because male mosquitoes do not contribute virus transmission. Mosquito population (*N_v_*) was determined by number of female mosquitoes per person (MPP), then set to MPP*N_h_*. Vector population was also divided into *S_v_* (susceptible), *E_v_* (exposed), and *I_v_* (infectious). Changes in each class of vector population are modeled by following differential equations:

(21)

$\frac{dE_{v}}{dt}=S_{v}a_{hv}b_{v}\frac{I_{h1}+I_{h2}+I_{h3}+I_{h4}+I_{h\_visit}}{N_{h}}-E_{v}(r_{eip}+d_{v})$ (22)

$\frac{dI_{v}}{dt}=E_{v}r_{eip}-I_{v}d_{v}$ (23)

where *Ih_visit* is the number of infected hosts who are temporary visiting the area but not included to the resident population. For strict mathematical consistency, *Ih_visit* should be added in the denominator; however as we set *Ih_visit* to a very small value (0.03-0.12) compared to total human population (100,000), it was omitted from the denominator.

To reflect the effect of temperature on vectors, we applied the temperature-dependent development rate (*r*) at temperature T (ºC) for the reproductive cycle of adult female mosquitoes (*r_fm_*) and viral replication in vectors (*r_eip_*) [9-11] :

$r=\frac{RHO25\left(T+273\right)/298\exp[(HA/R)\left\{(1/298)-1/(T+273)\right\}]}{1+\exp[(HH/R)\left\{1/(TH+273)-1/(T+273)\right\}]}$ (24)

where RHO25 is the development rate at 25ºC without any temperature inactivation of the enzyme, HA (cal･mol^-1^) is the enthalpy of activation of the reaction that is catalyzed by the critical enzyme, HH (cal･mol^-1^)is the enthalpy change with high-temperature inactivation of the enzyme and TH (ºC) is the temperature where a half of the enzyme is inactivated due to high temperature. R is the universal gas constant (1.987cal･mol^-1^･deg^-1^). Each RHO25, HA, HH and TH for the calculation of *r_fm_* and *r_eip_* were originally given by Focks *et al.*[9-11]. The length of gonotrophic cycle and the extrinsic incubation period (EIP) was the reciprocal of *r_fm_* and *r_eip_*, respectively.

Biting rate of the vector (*b_v_*) was determined by the number of blood meals on human beings per gonotrophic cycle (*B*).

　　　　　 $b_{v}=Br_{fm}$ (25)

As *Aedes aegypti* tends to take multiple blood meals per cycle [12], the value of *B* was set to 2.0 in our model. *Ae. aegypti* is an extremely domesticated and anthropophilic species, 100% of host preference on human beings was assumed.

**Table S**1: Parameter values for the simulations

| Parameter | Symbol | Value | Source |
| --- | --- | --- | --- |
| Host population | *N_h_* | 100,000 |  |
| Host death rate | *d_h_* | 0.000039 |  |
| Viral development rate in humans | *r_iip_* | 0.25 | [13,14] |
| Viral development rate in the vector bodies | *r_eip_* | Modeled by Equation 24  RHO25 0.080616  HA 15000  HH $6.203\times10^{21}$ TH $-2.176\times10^{30}$ | [11] |
| Mosquito density per person | MPP | Modeled |  |
| Vector population | *N_v_* | MPP*N_h_* |  |
| Vector death rate | *d_v_* | 0.11 | [15,16] |
| Recovery rate of humans | *r_recovery_* | 0.2 | [5,17] |
| Duration of cross immunity | *T_cross_* | 60 days | [1] |
| Transmission probability | | | [18] |
| Vector to host | *a_vh_* | 0.75 |
| Host to vector | *a_hv_* | 0.75 |
| Number of serotypes | *n* | 1, 2, 4 |  |
| Visiting infectious host | *Ih_visit* | 0.03 *n* | [11] |
| Proportion of infectious vector | *q_1_–q_4_* | *q_1_* = 1  *q_2_* = 0.5 with two serotypes  *q_2_* = 0.75 with four serotypes  *q_3_* = 0.5  *q_4_* = 0.25 | [8] |
| Number of blood meals per cycle | *B* | 2.0 | [12] |
| Biting rate | *b_v_* | *Br_fm_* |  |
| Development rate of adult female mosquitoes | *r_fm_* | Modeled by Equation 24  RHO25 0.21552  HA 15,725.23  HH 1,756,481.07  TH 39.0* | [9,10] |
| Population immunity | *p_i_* | 0-0.8 |  |

* modified by the authors on the basis of Christophers [19].

REFERENCES

1. Sabin AB (1952) Research on dengue during World War II. Am J Trop Med Hyg 1: 30-50.
2. Scherer WF, Breakenridge FA, Dickerman RW (1972) Cross-protection studies and search for subclinical disease in new world monkeys infected sequentially with different immunologic types of dengue viruses. Am J Epidemiol 95: 67.
3. Gibbons RV, Kalanarooj S, Jarman RG, Nisalak A, Vaughn DW, et al. (2007) Analysis of repeat hospital admissions for dengue to estimate the frequency of third or fourth dengue infections resulting in admissions and dengue hemorrhagic fever, and serotype sequences. Am J Trop Med Hyg 77: 910.
4. McLean DM, Clarke AM, Coleman JC, Montalbetti CA, Skidmore AG, et al. (1974) Vector capability of *Aedes aegypti* mosquitoes for California encephalitis and dengue viruses at various temperatures. Can J Microbiol 20: 255-262.
5. Gubler DJ, Suharyono W, Tan R, Abidin M, Sie A (1981) Viraemia in patients with naturally acquired dengue infection. Bull World Health Organ 59: 623-630.
6. Watts DM, Burke DS, Harrison BA, Whitmire RE, Nisalak A (1987) Effect of temperature on the vector efficiency of *Aedes aegypti* for dengue 2 virus. Am J Trop Med Hyg 36: 143-152.
7. Salazar MI, Richardson JH, S nchez-Vargas I, Olson KE, Beaty BJ (2007) Dengue virus type 2: replication and tropisms in orally infected *Aedes aegypti* mosquitoes. BMC microbiol 7: 9.
8. Luz PM, Vanni T, Medlock J, Paltiel AD, Galvani AP (2011) Dengue vector control strategies in an urban setting: an economic modelling assessment. The Lancet 377: 1673-1680.
9. Focks DA, Haile DG, Daniels E, Mount GA (1993) Dynamic life table model for *Aedes aegypti* (Diptera: Culicidae): analysis of the literature and model development. J Med Entomol 30: 1003-1017.
10. Focks DA, Haile DG, Daniels E, Mount GA (1993) Dynamic life table model for *Aedes aegypti* (diptera: Culicidae): simulation results and validation. J Med Entomol 30: 1018-1028.
11. Focks DA, Daniels E, Haile DG, Keesling JE (1995) A simulation model of the epidemiology of urban dengue fever: literature analysis, model development, preliminary validation, and samples of simulation results. Am J Trop Med Hyg 53: 489-506.
12. Scott TW, Clark GG, Lorenz LH, Amerasinghe PH, Reiter P, et al. (1993) Detection of multiple blood feeding in *Aedes aegypti* (Diptera: Culicidae) during a single gonotrophic cycle using a histologic technique. J Med Entomol 30: 94-99.
13. Kuno G (1995) Review of the factors modulating dengue transmission. Epidemiologic reviews 17: 321-335.
14. Gubler DJ (1998) Dengue and dengue hemorrhagic fever. Clin Microbiol Rev 11: 480-496.
15. Patz JA, Martens W, Focks DA, Jetten TH (1998) Dengue fever epidemic potential as projected by general circulation models of global climate change. Environ Health Perspect 106: 147.
16. Vaughan JA, Focks DA, Turell MJ (2009) Simulation Models Examining the Effect of *Brugian Filariasis* on Dengue Epidemics. Am J Trop Med Hyg 80: 44.
17. Nishiura H, Halstead SB (2007) Natural history of dengue virus (DENV)-1 and DENV-4 infections: reanalysis of classic studies. J Infect Dis 195: 1007-1013.
18. Newton EA, Reiter P (1992) A model of the transmission of dengue fever with an evaluation of the impact of ultra-low volume (ULV) insecticide applications on dengue epidemics. Am J Trop Med Hyg 47: 709-720.
19. Christophers S (1960) *Aedes aegypti* (L.) The Yellow Fever Mosquito: Its Life History, Bionomics and Structure. Cambridge: Cambridge University Press. 475 p.
